# Low-intensity pulsed ultrasound: Fracture healing

**DOI:** 10.4103/0019-5413.50847

**Published:** 2009

**Authors:** Raman Mundi, Stephen Petis, Roopinder Kaloty, Vijay Shetty, Mohit Bhandari

**Affiliations:** Division of Orthopaedic Surgery, McMaster University, Hamilton, Ontario, Canada; 1 Consultant Orthopaedic Surgeon, Dr. LH Hiranandani Hospital, Pawai, Mumbai, India

**Keywords:** Ultrasound, fracture healing, systematic review, randomized controlled trials

## Abstract

Annually, millions of people across the world are inflicted with bone fracture injuries. Untimely healing is a significant burden in terms of socioeconomic costs, personal costs, and patients' quality of life. Low-intensity pulsed ultrasound (LIPUS) has gained much attention as a potential adjunctive therapy for accelerating fresh fracture healing, but its efficacy remains controversial. This paper is presented in two parts a literature review followed by a systematic review. The literature review highlights the physiology of fracture healing and the influence LIPUS exerts on cells and molecules involved in this healing process. In part two, we present a systematic review of randomized controlled trials (RCTs) assessing the clinical effectiveness of LIPUS in accelerating the time to fracture healing. The electronic databases we searched for the systematic review are as follows: MEDLINE (from 1996 to November 2008), EMBASE (from 1996 to November 2008), and Healthstar (from 1966 to October 2008). A two-step screening process was used to assess the eligibility of studies yielded by our search. The first step was a review of titles and abstracts for the selection of studies that met the following criteria: (i) inclusion of skeletally mature patients with a fresh fracture, (ii) a minimum of two treatment arms with at least one arm receiving LIPUS treatment and another arm receiving placebo, (iii) random allocation of patients to the different treatment arms, (iv) radiological assessment of time to fracture healing, and (v) publication in the English language. In the second step, selected articles were reviewed in full text. Eligible trials were all scored independently by two reviewers for methodological reporting quality using the 15-item CLEAR NPT checklist (Checklist to Evaluate the Report of a Nonpharmacological Trial). We identified a total of seventy seven studies, nine of which met our inclusion criteria after the initial screening. Of these nine trials, seven were included for the final review. The types of fractures studied among these seven trials included lateral malleolar, radial, and tibial fractures. Three of the seven trials found that LIPUS significantly reduces healing time compared to placebo, whereas the other four did not find a statistically significant difference. There is a substantial level of inconsistency in the findings of several RCTs evaluating the efficacy of LIPUS as an adjunct for fracture healing. Although LIPUS has proven to be effective in certain trials for accelerating fracture healing, no definitive statement can be made regarding its universal use for all fracture types and methods of fracture care. Future high-quality RCTs with larger sample sizes may help to elucidate the specific indications that warrant or dismiss the need for LIPUS therapy.

## INTRODUCTION

Bone fractures represent a global medical challenge for health care administrations, orthopedic care providers, and patients alike, as millions of people across the world are inflicted with these injuries annually.^1^

Fractures that heal ideally without complications can take months to heal completely.^2^ During this time of treatment and recovery, there is significant burden in terms of socioeconomic costs, personal costs, and patient quality of life.^2^–^4^ To make matters more troublesome, not all fractures heal at an “ideal” rate, and these delayed unions or nonunions further compound the costs and personal hardships associated with fracture care and recovery.^2^,^4^ Infact, of the estimated 6.2 million fractures occurring annually in the United States, between 5% and 10% exhibit either delayed healing or nonunion.^1^

Due to the significant repercussions of untimely fracture healing, substantial research has sought to elucidate the effectiveness of adjunctive therapies for accelerating fresh fracture healing. One such treatment that has gained much attention is ultrasound therapy.

Ultrasound is a source of mechanical energy delivered as acoustic pressure waves beyond the range of human hearing. It has a variety of medical applications, ranging from a diagnostic tool to a therapeutic agent. Typically, at low intensities (0.5–50 mW/cm^2^), ultrasound serves a diagnostic purpose, whereas at higher intensities (0.2-100 W/cm^2^), its role becomes more therapeutic by means of generating heat energy.^5^ Although early studies found high-intensity ultrasound to delay bone healing, more recent studies using low-intensity pulsed ultrasound (LIPUS) in the diagnostic range have demonstrated more favorable effects.^4^ The role of ultrasound therapy in fresh fracture healing remains controversial, however, both in terms of its mechanism of action and its efficacy in the clinical setting.

The objectives of this present study were two-fold. In the first phase of this study, we carried out a literature review to illustrate the mechanisms by which ultrasound therapy has been proposed to accelerate bone healing. In the second phase of this study, we conducted a systematic review of randomized controlled trials (RCTs) evaluating the clinical effectiveness of LIPUS in accelerating bone healing.

## LITERATURE REVIEW

### Physiology of fracture healing

The healing process of a fractured bone involves four steps: inflammation, soft callus formation, hard callus formation, and bone remodeling. Following injury, the initial inflammatory phase involves damage to blood vessels, periosteal tissue, osteon units, and perforating canals.^6^ The damaged blood vessels result in the formation of a fracture hematoma, which functions to occlude blood flow to the site of injury. Such occlusion of blood flow results in necrosis of the bone and subsequently, the release of inflammatory cytokines that initiate angiogenesis and induce osteoclastic and macrophagic activity.^1^,^6^ Angiogenesis is critical in delivering adequate oxygen, nutrients, and cells to the site of injury to promote healing, whereas osteoclasts and macrophages function in the removal of dead tissue and cellular debris.^7^

Following inflammation is the genesis of a soft or fibrocartilaginous callus. Angiogenesis promotes the delivery of osteogenic cells and fibroblasts to the site of injury, resulting in the formation of an initial “procallus”.^6^,^7^ The fibroblasts secrete collagen to temporarily connect the broken ends of the bone, and the osteogenic cells differentiate in the avascular environment into chondroblasts. The chondroblasts subsequently deposit fibrocartilage, which converts the procallus into the characteristic soft callus in the process of fracture healing.^6^,^8^

Fracture healing continues with the evolution of the soft callus into a hard, bony callus. This process is initiated by the differentiation of osteogenic cells into osteoblasts in the well-vascularized bone tissue. The osteoblasts initiate intramembranous ossification, replacing the soft callus with a trabeculae network of bone connecting the developing and necrotic bone fragments.^1^,^6^ This is accomplished via the release of sequestered organic bone matrix and calcium salts from within the osteoblasts.^9^

Bone remodeling is the final phase of fracture healing. Osteoclasts continue to remove necrotic bony tissue to accommodate space for the newly formed bone.^6^ Simultaneously, osteoblasts replace the trabeculae bone with compact bone through endochondral ossification.^1^ The only footprint left of bone repair is a thickened area on the surface of the bone lacking the presence of a fibrotic scar.^6^,^7^

A substantial number of studies are examining various interventions to enhance the aforementioned fracture healing process. Stem cell research, gene therapy, drug administration, transfusions, and ultrasonography are just some of the current areas of research.^10^ The use of ultrasound as a therapeutic tool is of particular interest, providing a noninvasive physical stimulus that may enhance fracture healing.

### Ultrasound: What is it and what does it do?

A fundamental understanding of ultrasound (US) functionality is essential to understanding its role in the physiology of fracture healing. Ultrasound is a modality that applies transcutaneous acoustic energy for diagnostic and therapeutic purposes. Sound waves produced by a piezoelectric crystal are transmitted through various body tissues to induce a number of physiologic changes implicated in tissue healing.^11^,^12^ The proportion of sound waves absorbed by a specific tissue is directly related to that tissue's density. Bone typically possesses the densest tissue in a given area, allowing for the use of US waves to effectively target areas where bony abnormalities may exist.^13^

LIPUS, in particular, serves as a potential noninvasive therapeutic toward fracture healing.^2^ The waves administered by LIPUS induce micromechanical stress in the fracture site, culminating in the stimulation of various molecular and cellular responses involved in fracture healing.^1^ The beneficial osteogenic and angiogenic effects observed after LIPUS administration are largely nonthermal (< 1°C), and rather mechanical in nature. The operating parameters used to achieve these benefits include a 30-mW/cm^2^ intensity, 1.5-MHz frequency repeated at 1 kHz, and a pulse width of 200 μs administered for 20 minutes each day.^1^,^4^

### Ultrasound: How does it exert mechanical stress?

Two proposed mechanisms exist suggesting how LIPUS induces micromechanical stress in bony tissues: (i)displacement of the fractured ends and (ii) cavitation.

The first mechanism involves the motion caused at both ends of a fractured bone by the pulsed waves of LIPUS. One body of research suggests that this motion occurs on a nanometric scale (displacements of 0.15–0.55 nm) to stimulate molecular and cellular pathways involved in healing.^14^ Claes and Willie^1^ suggest that LIPUS results in a micromotion displacement (0.5–2 mm) at the borders of soft and hard tissues (soft and hard calluses, respectively), producing a more salient mechanical stimulus to the integrin mechanoreceptors involved in cellular signaling and osteogenic differentiation. It remains uncertain as to which of these displacement mechanisms dominate in enhancing the fracture healing process.

The other proposed mechanism for LIPUS-induced micromechanical stress to bony tissues involves cavitation and acoustic streaming. This thought endorses the idea that pulsating sound waves from LIPUS permit the accumulation of gas bubbles within cells and tissues, creating a cavity to support acoustic streaming. These bubbles can remain either stable (stable cavitation) or unstable (unstable cavitation). Stable cavitation allows for acoustic streaming, which causes a slight turbulence or circular flow of tissue fluids as sound waves maneuver around the gas bubbles.^15^ This process culminates in increased cell permeability, causing a subsequent rise in blood pressure at the site of injury. The elevated blood pressure accelerates healing by enhancing gas exchange and nutrient delivery.^16^ Unstable cavitation results in bursting bubbles, with the resultant energy stimulating surrounding tissues.^17^

### Ultrasound: The effect of mechanical stress on molecules and cells

Several studies have demonstrated the potential for LIPUS to accelerate fracture healing by altering molecular and cellular mechanisms involved in each stage of the healing process.

#### The role of integrins

Integrins play a particularly important role in modulating cellular signaling involved in fracture healing.^14^ Acting as mechanoreceptors, integrin proteins react to vibrations and pressure changes created by LIPUS in the cellular environment. These mechanical stimuli increase focal adhesions (integrin clusters) on fibroblasts and upregulate integrin mRNA expression in osteoblasts.^18^,^19^ These changes enhance the respective cells' sensitivity to motion in the environment and increase their intracellular signaling capacity.^14^ The most significant outcome of induced intracellular signaling in osteoblasts is the heightened activation of the cyclooxygenase-2 (COX-2) enzyme.^20^ This results in an increased production of prostaglandin E2, a leukotriene critical to effective mineralization during endochondral ossification of the soft callus.^21^

#### Effect on endochondral ossification

Alizarin red staining has verified an increase in calcium nodule formation within the osteoblasts following LIPUS.^22^ This results in increased stiffness and thickness in the bony callus and healed fracture.^1^

LIPUS-enhanced endochondral ossification also results in a larger area and greater extent of bony callus formation by augmented mineral deposition. These changes are demonstrated by a smaller fracture gap following LIPUS, as well as increased cortical bone mass.^1^ Increased bone volume, cortical bone thickness, and mineral apposition also suggest that LIPUS may enhance the anabolic activity of osteoblasts, especially early in their differentiation as a lineage.^12^ Studies have also demonstrated increased phagocytosis during inflammation, accelerated callus formation, and catalyzed mineralization following LIPUS administration.^23^,^24^

#### Effect on cells involved in fracture healing

LIPUS also plays a role in stimulating differentiation of some of the cells involved in fracture healing. These include chondroblasts, mesenchymal cells, fibroblasts, and osteoblasts.^1^ LIPUS increases the expression of aggrecan, a structural macromolecule of cartilage which acts as a potent stimulant of chondrogenesis. The increased concentration of aggrecan leads to accelerated chondroblast differentiation into chondrocytes.^25^ Having more chondrocytes at the site of injury results in an increase in the release of chondroitin sulfate, an essential component in supporting cartilaginous and bony structures.^6^,^8^ Also, due to the aforementioned cavitation mechanism, LIPUS increases blood pressure at the fracture site due to an increase in vascular permeability.^17^ This increase in hydrostatic pressure has been linked to the increased differentiation of mesenchymal stem cells into chondroblasts, which may also enhance the development of the fibrocartilaginous callous.^6^,^26^

LIPUS also causes increased expression of early osteogenic genes, including osteonectin, osteopontin, and insulin growth factor-1. These play a crucial role in ensuring proper osteoblast differentiation.^27^,^28^ Osteoprogenitor cells from the bone marrow may also differentiate into osteoblasts at an increased rate by detecting the LIPUS-induced increase in local blood pressure via membranous integrin proteins.^14^,^18^,^19^,^26^ Alternatively, the increased hydrostatic pressures exerted by LIPUS may reduce osteoclast differentiation from progenitor cells distributed to the fracture site via angiogenesis.^26^ Interestingly, the hemodynamic shear stress induced by increased blood pressure and subsequent increased fluid flow, as well as increased fluid turbulence caused by the modality sound waves at the fracture site may act as a prominent stimulant in the recruitment of osteoprogenitor cells from the bone marrow, thus enhancing bone healing and remodeling.^9^,^15^,^26^

As mentioned previously, LIPUS causes very small changes in tissue temperature.^1^ Welgus *et al.*^29^ suggest that these small alterations in tissue temperature may stimulate interstitial collagenase or collagenase-1-fibroblastic enzymes that assist in soft callus formation and bridging the fractured ends of the bone together. Lastly, angiogenesis is enhanced by LIPUS through an increase in mRNA expression and production of vascular endothelial growth factor by both human osteoblasts and periosteal cells.^30^,^31^

Despite the substantial cellular research that has strived to reveal the mechanisms by which LIPUS therapy enhances fracture healing, uncertainty regarding these mechanisms still persists. Nevertheless, there is clinical evidence-in addition to the aforementioned laboratory evidence-that LIPUS induces changes that may facilitate and accelerate the union of broken bones. Several RCTs have evaluated the utility of LIPUS as a fracture healing therapy, as discussed in the following systematic review.

## SYSTEMATIC REVIEW

### Objective

We conducted a systematic review of RCTs to evaluate whether LIPUS accelerates healing time of fractures sustained in skeletally mature patients.

### Methods

#### Search strategy

One investigator systematically searched through several electronic databases to identify and retrieve relevant randomized trials published in the English language from 1966 to November 2008. Specifically, three databases were searched: MEDLINE (from 1996 to November 2008), EMBASE (from 1996 to November 2008), and Healthstar (from 1966 to October 2008). For all databases, we used the following search terms: (i) “ultras*” (ii) “fracture healing” and (iii) “random*.” The asterisks (“*”) were utilized to improve the sensitivity of our search strategy, as any word with the letters preceding the asterisk would be incorporated in the search. For instance, the term “random*” would yield papers utilizing terms such as “randomized,” “randomly,” or simply just “random.” Thus, by incorporating these asterisks, we strived to limit the inadvertent exclusion of potentially relevant trials.

#### Eligibility criteria

Eligibility criteria for the inclusion of trials in the current systematic review were established a priori-that is, before the search strategy was conceived and implemented. Potentially relevant trials eligible for inclusion had to meet the following criteria: (i) inclusion of skeletally mature patients with a fresh fracture, (ii) a minimum of two treatment arms with at least one arm receiving LIPUS treatment and another arm receiving placebo, (iii) random allocation of patients to the different treatment arms, (iv) radiological assessment of time to fracture healing, and (v) publication in the English language. One investigator performed a two-step screening process to assess the eligibility of all studies yielded by the aforementioned search strategy. The first step entailed the review of all titles and abstracts. Only those studies that met the aforementioned five inclusion criteria based on their abstracts were selected for the second screening step, which consisted of a full-text review for eligibility criteria.

#### Methodological quality

Eligible trials were all assessed for their reporting quality as determined by the CLEAR NPT checklist (Checklist to Evaluate the Report of a Nonpharmacological Trial). The CLEAR NPT is a 15-item checklist (10 primary items and 5 subitems) that evaluates the reporting quality of nonpharmacological randomized trials. Briefly, the main items on this checklist assess (i) generation of allocation sequence, (ii) allocation concealment, (iii) details of intervention, (iv) care provider skill, (v) participant adherence, (vi) blinding of participants, (vii) blinding of care providers, (viii) blinding of outcome assessors, (ix) follow-up schedule, and (x) utilization of the intention-to-treat principle. The questions on this checklist are typically answered as “yes” “no” or “unclear.”

Two investigators independently applied this checklist to all eligible trials, and their responses were compared. Any discrepancies were resolved by joint review of the necessary trials until consensus was reached. To enhance our interrater reliability, we utilized a supplemental scoring guideline that provided an objective approach to answering each item on the checklist.

#### Data extraction

The outcome of interest for the purpose of this review was “time to healing” of fresh fractures. Data on the time to healing, as determined solely by radiographical evidence (bridging of three or four cortices), was the preferred outcome measure and extracted from studies that reported such data. If a study did not use radiographic assessment as the sole measure of fracture healing and this information was not available, days to fracture healing was recorded based on that particular study's defining criteria (e.g., combination of clinical stability with no pain and radiological healing).

Additional information extracted from the studies included details of the ultrasound and control intervention, patient data (sample size, age), and fracture characteristics (type, fracture treatment).

## RESULTS

**Figure 1:**
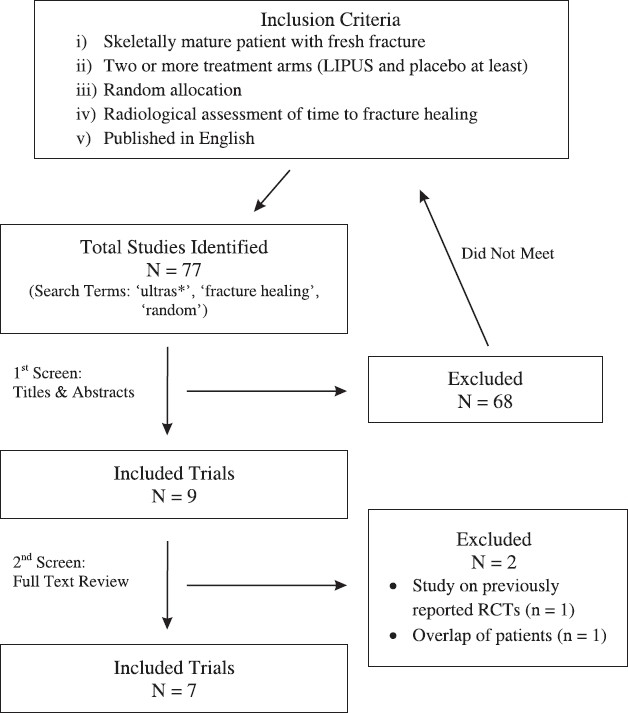
Search strategy and screening

We identified a total of seventy seven studies, of which Nine met our inclusion criteria after initial screening of the titles and abstracts. Of these nine trials, seven were included for the final review [Figure 1]. Among the two excluded, one^32^ was a report on previously published data of two RCTs (both of which are included in this review). The second study^33^ consisted of patients who were also analyzed in a different paper^34^ written by the same lead author. This overlap of patients in the two papers was verified by the author, and only the paper with the longer recruitment period and larger sample size was included in this review.

To ensure that our search did not inadvertently omit relevant trials, we cross-referenced our search results with two other systematic reviews assessing RCTs evaluating the effect of LIPUS on fracture healing. In the review by Busse *et al.*, (2002) published their search strategy included five electronic databases, a hand search of seven journals, as well as contacting experts in the field.^4^ Incorporating studies of any language, their search strategy yielded six trials, of which our study overlooked one trial by Emami *et al*.^35^ More recently, Walker and associates^36^ performed a review in 2007 and incorporated five randomized trials, of which our search overlooked the same trial by Emami *et al*.^35^ However, this paper was excluded from the final analysis by Busse *et al.* as they realized that it reported data on the same patient population as a second report by Emami *et al.*^37^ -the latter being included in our current review. Furthermore, of the seven trials included in our review, three trials were not assessed by either of these other reviews.

### Quality assessment with CLEAR NPT

The results of the reporting quality assessment are presented in Table 1. Three (43%) of the seven studies explicitly reported an appropriate means of randomizing their patients, and of these three trials, two trials (29%) concealed patient allocation prior to randomization. The remaining trials did not adequately carry out, or were unclear in reporting, randomization, and allocation concealment. All seven (100%) studies provided sufficient details on the treatment protocol and had the same follow-up schedules for both treatment arms.

**Table 1:** Quality of reporting in RCTs assessed with the CLEAR NPT

	Yes	No	Unclear	Not appl.
Was the generation of allocation sequences adequate?	3	1	3	
Was the treatment allocation concealed?	2	1	4	
Were details of the intervention administered to each group made available?	7	0	0	
Were care providers' experience or skill in each arm appropriate?	–	–	–	7*
Was participant adherence assessed quantitatively?	4	0	3†	
Were participants adequately blinded?	7	0	0	
Were care providers or persons caring for the participants adequately blinded?	6	0	1‡	
Were outcome assessors adequately blinded to assess the primary outcomes?	5	0	2‡	
Was the follow-up schedule the same in each group?	7	0	0	
Were the main outcomes analyzed according to the intention-to-treat principle?	4	2	1	

* Ultrasound therapy can be self-administered and is not influenced by care provider's skill.

† Although these trials stated their qualitative methods for assessing adherence, they failed to report this data for one or both treatment arms.

‡ Stated double-blinded, but did not specify which parties in particular were blinded other than patients.

Previous work has demonstrated that the term “double-blinded” has an inconsistent interpretation, and often describes blinding of various combinations of parties involved in a research trial (i.e., patients and care providers, patients and outcome assessors, etc.).^38^ Therefore, despite all seven trials in our review stating that their studies were “double-blinded,” we required the report to make clear exactly who was blinded. In all seven (100%) studies, patients were blinded as to whether they were receiving ultrasound or placebo. In six (86%) studies, the treatment (ultrasound or placebo) was self-administered and the ‘care providers’ (i.e., patients) were blinded, whereas the outcome assessor was blinded in five (71%) of the seven studies. In the remaining trials, it was unclear whether the care providers and outcome assessors were blinded or unblinded.

### Study characteristics

All seven studies randomized their patients into two treatment arms: an ultrasound group and a placebo group. Furthermore, in all studies, the treatment group received 20 minutes of daily ultrasound therapy from the Sonic Accelerated Fracture Healing System (Exogen, Piscataway, NJ).^39^ Six studies specified the characteristics of the ultrasound signal utilized. All six used an ultrasound signal consisting of a burst width of 200 *μ*s containing 1.5 MHZ sine waves, with a repetition rate of 1 kHz, and a spatial average temporal intensity of 30 mW/cm^2^.^5^,^34^,^37^,^40^–^42^ In terms of the control group, all seven studies treated patients for 20 minutes daily with a sham ultrasound unit.

The seven trials enrolled a total of 262 patients, and the number of patients recruited in each trial ranged from 22 to 66. There were a total of 283 fractures treated and the sample size treated for each trial ranged from 22 to 67 (select patients had multiple fractures). Among the five trials that provided information on age distribution, the patients enrolled in these trials ranged from 17 to 73 years of age [Table 2].

**Table 2:** Summary of the RCTs comparing LIPUS to placebo

Trial	Fracture treatment	LIPUS and placebo therapy duration (onset)	Sample size (no. of fractures)		Mean age (range)		CLEAR NPT†
					
			LIPUS	Placebo	LIPUS	Placebo	
Malleolar Handolin *et al*., 2005^34^	SR-PLLA screw + 6-week immobiliz.	6 weeks (3rd postop. week)	15	15	41.4 (19–65)	39.4 (18–59)	6/9
Handolin *et al*., 2005^40^	SR-PLLA screw + 6-week immobiliz.	6 weeks (3rd postop. week)	11	11	37.5 (18–54)	45.5 (26–59)	6/9
Radial Kristiansen *et al*., 1997^5^	Closed reduction + cast immobiliz.	10 weeks (<7 days of injury)	30	31	54 (N/A)	58 (N/A)	9/9
Tibial Heckman *et al*., 1994^41^	Closed reduction + cast immobiliz	20 weeks or sufficient healing (<7 days of injury)	33	34	36 (N/A)	31 (N/A)	8/9
Leung *et al*., 2005^42^	(i) Reamed intramed. nail, or	90 days (stabilized patient)	16	14	‡	‡	5/9
	(ii) External fixation						
Emami *et al*., 1999^37^	Reamed and locked intramed. nail	75 days (<3 postop. days)	15	15	39.9 (21–73)	36.5 (19–57)	7/9
Rue *et al*., 2004^43^	Protected weight bearing + exercise + calcium + vitamin	Clinical and radiographic healing (average 29-day delay from symptoms)	§	§	18.6 (18–20)	18.4 (17–20)	4/9

† Of the 10 main items on the CLEAR NPT, only 9 are applicable (item 4 regarding care provider experience/skill is not applicable for the current trials). No. of items recorded as “yes”/total no. of items.

‡ Data are only given for combined groups (mean age = 35.3, Range = 22–61).

§ There were a total of 43 fractures (14 patients treated with LIPUS and 12 with placebo); however, the number of fractures per treatment arm was not provided. The outcomes measured were based on the patient, as opposed to the individual fractures.

### Fractures, treatments, and time to fracture healing

Several types of fractures and treatment modalities were investigated among the seven trials [Table 2]. The time to fracture healing for each trial is presented in Table 3.

**Table 3:** Time to fracture healing-LIPUS versus placebo

Trial	Radiographic definition of fracture healing*	Mean days to fracture healing or fraction of patients healed (no. of weeks)		Statistical significance (*P* value)
			
		LIPUS	Placebo	
Malleolar	Callus formation	14/15	12/15	No
Handolin *et al*., 2005^34^		12th postop. week)	(12th postop. week)	
Handolin *et al*., 2005^40^	Callus formation	8/10	9/11	No
		(12th postop. week)	(12th postop. week)	
Radial Kristiansen *et al*., 199^75^	Bridging of 4 cortices	61 ± 3 days	98 ± 5 days	Yes *P* < 0.0001
Tibial Heckman *et al*., 1994^41^	Bridging of 4 cortices Bridging of 3 of 4 cortices	114 ± 7.5 days	182 ± 15.8 days	Yes *P* = 0.0002
Leung *et al*., 2005^42^	Bridging 3 of 4 cortices	11.5 ± 3.0 weeks	20 ± 4.4 weeks	Yes *P* < 0.05
Emami *et al*., 1999^37^	“Signs of healing like cortical thickening”	155 ± 22 days	129 ± 12 days	No
Rue *et al*., 2004^43^		56.2 ± 19.6 days	55.8 ± 15.5 days	No

* Although individual trials may have reported other criteria for fracture healing, signs of radiographic healing were of interest for the current review.

### Lateral malleolar fractures

Handolin and colleagues carried out two RCTs to assess the effect of LIPUS on the healing of lateral malleolar fractures treated with a self-reinforced poly-l-lactide (SR-PLLA) bioabsorbable screw.^34^,^40^

In the first of these studies, all 30 patients underwent fracture fixation with the bioabsorbable screw followed by immobilization for six weeks in a removable cast. After the second postoperative week, 15 patients received ultrasound therapy and 15 received placebo for a duration of six weeks. At the final followup on the twelfth postoperative week, radiographic assessment of time to fracture healing-as measured by callus formation-revealed no significant difference between the two groups (14/15 in LIPUS vs. 12/15 in placebo had demonstrated callus formation on posterior cortex of fibula).^34^

In their second trial, they recruited 22 patients and followed the same protocol in terms of fracture fixation, immobilization, as well as onset and duration of ultrasound or placebo therapy. There was no difference between the groups with respect to callus formation at the twelfth postoperative week (8/10 in LIPUS and 9/11 in placebo group had developed a callus). In this trial, patients were also assessed with multidetector computed tomography (MDCT) to evaluate endosteal bone healing, assessed as the portion of 'united' to ‘nonunited’ fracture line. At nine postoperative weeks, no difference was found between the groups (42.5% LIPUS vs. 38.8% placebo, *P* = 0.812).^40^

### Radial fractures

In a multicenter trial by Kristiansen and associates, the effect of LIPUS on the healing rate of dorsally angulated metaphyseal fractures of the distal radius was evaluated. 60 patients (61 fractures) were all treated with closed reduction and immobilization with a below-the-elbow cast. Within seven days of suffering the fracture, all patients were treated and began receiving ultrasound (n = 30) or placebo (n = 31) therapy and continued to receive therapy for a total of ten weeks. Radiographic assessment for complete bridging of all four cortices (dorsal, volar, radial, and ulnar) revealed a significant reduction in the time to fracture healing between the LIPUS and placebo group, in favor of the LIPUS group (61 ± 3 days LIPUS vs. 98 ± 5 days placebo, difference of 37 days, *P* < 0.0001). This study also demonstrated that the significant effect of ultrasound on healing time persisted after patients were stratified for age (≤49 vs. ≥50), gender, and the degree of volar angulation before reduction (≤ −9° vs. ≥ −10°).^5^

### Tibial fractures

Several studies have addressed the use of LIPUS for various types of tibial fractures with different treatment modalities.

#### Significant results

LIPUS therapy has been shown to significantly decrease healing time in a study of 67 closed or grade I open diaphyseal fractures of the tibia, treated by closed reduction and cast immobilization. The time to bridging of all four cortices was significantly less for the 33 fractures treated with LIPUS than for the 34 treated with placebo (114 ± 7.5 days LIPUS vs. 182 ± 15.8 days placebo, *P* = 0.0002). It was also demonstrated that the location of the fracture-proximal, middle, or distal-did not influence the efficacy of LIPUS therapy.^41^

Further endorsing the use of LIPUS as an effective therapy for the treatment of tibial fractures are the results of Leung *et al*.'s study. In their study of 30 complex open and closed tibial fractures, patients were treated either with reamed intramedullary nailing (diaphyseal closed and Gustillo I and II open fractures) or external fixation (metaphyseal and Gustillo IIIA fractures). The time to healing between the LIPUS (n = 16) and placebo group (n = 14)-both therapies commencing upon stabilization of the patient and continuing for ninety days-was significantly reduced in the LIPUS group as determined by the appearance of a third callus (11.5 ± 3 weeks for LIPUS vs. 20 ± 4.4 weeks for placebo, *P* < 0.05). Both the LIPUS and placebo groups were comparable for the number of open and closed fractures.^42^

#### Nonsignificant results

In contrast to the aforementioned studies, other studies have demonstrated conflicting results regarding the treatment of tibial fractures with LIPUS therapy.

Emami *et al*. studied a sample of 30 patients with tibial shaft fractures treated with a reamed and locked intramedullary nail. Within three postoperative days, half the patients were treated with LIPUS and the other half with placebo, for a duration of seventy-five days. There was no significant difference in the time to radiographic healing (bridging of three of four cortices) between the two groups (155 ± 22 days for LIPUS vs. 129 ± 12 days for placebo).^37^

In a study of 26 young adults (43 fractures) undergoing a rigorous six-week training program at a US Naval Academy, the effect of LIPUS on the healing time of tibial stress fractures was assessed. All of these physically fit recruits were treated with standard care consisting of protected weight-bearing, alternative aerobic exercise, calcium supplementation, and daily multivitamin. In addition, 14 received ultrasound therapy whereas 12 received placebo until clinical stability (no pain upon palpitation + painless single-leg hop) as well as radiographic “signs” of healing. There was no difference between the two groups in terms of fracture healing, as the average time from onset of symptoms to return to training was fifty-six days in both groups.^43^

## DISCUSSION

The process of fracture healing occurs in four consecutive stages: inflammation, soft callus formation, hard callus formation, and bone remodeling. The findings of several laboratory studies suggest that the use of LIPUS can accelerate the healing process by influencing all four stages of fracture healing. Specifically, the mechanical stresses resulting from the emitted acoustic pressure waves serve to manipulate the expression and functioning of various cells and molecules involved in the healing process.

Clinically, several randomized trials have assessed the effectiveness of LIPUS in enhancing the rate of fresh fracture healing. Our current systematic review highlights the conflicting findings surrounding this body of research. Of the seven trials included in our review, three trials found LIPUS to significantly reduce healing time whereas four did not. However, there is one noticeable difference between the trials with significant and nonsignificant findings. In all three trials with significant findings, ultrasound or placebo therapy commenced early-either within seven days of injury or upon stabilization of the patient.^5^,^41^,^42^ In contrast, Handolin *et al*. did not treat patients with ultrasound or placebo therapy until the third postoperative week, and in the Naval Academy study, the average time between symptom onset and adjunctive therapy was twenty-nine days.^34^,^40^,^43^ The one study that does not fit this description is the study by Emami *et al.*^37^ Despite early treatment with ultrasound or placebo therapy, they demonstrated nonsignificant findings. A potential explanation for these findings is that the intramedullary nail may create a construct that is too stable for the US signal to exert mechanical stresses.^2^

Furthermore, several studies had enrolled small sample sizes, thus increasing the risk of type II error (detecting no difference when a meaningful difference actually exists). Thus, the inability to detect a significant difference by these trials should not immediately be deemed as no possible effect of LIPUS on fracture healing,

With global incidence rates in the millions each year, coupled with the associated personal and economic costs, bone fractures are a true medical challenge.^1^–^4^ Implementing adjunctive therapies to enhance fracture healing is of utmost importance. Currently, conflicting results from high-quality randomized trials suggest that LIPUS therapy may accelerate fracture healing, although no universally definitive statement can be made. LIPUS therapy appears to have varying influences on bone healing depending on the onset of therapy, fracture type, and the approach to fracture care (i.e., operative or nonoperative), among other potential factors. Further randomized trials, with adequate sample sizes and sound methodological rigor (blinding, allocation concealment, etc.) are warranted to elucidate the clinical circumstances in which LIPUS is truly efficacious and the optimal approach to delivering this treatment modality.
